# First molecular detection and characterization of herpesvirus and poxvirus in a Pacific walrus (*Odobenus rosmarus divergens*)

**DOI:** 10.1186/s12917-014-0308-2

**Published:** 2014-12-21

**Authors:** Mar Melero, Daniel García-Párraga, Juan Manuel Corpa, Joaquín Ortega, Consuelo Rubio-Guerri, José Luis Crespo, Belén Rivera-Arroyo, José Manuel Sánchez-Vizcaíno

**Affiliations:** VISAVET Center, Veterinary School, Complutense University of Madrid, 28040 ᅟ, Madrid Spain; Veterinary Services, Oceanografic, Parques Reunidos Valencia, Ciudad de las Artes y las Ciencias, 46013 ᅟ, Valencia Spain; Biomedical Sciences Research Institute (PASAPTA-Pathology Group), Veterinary School, Universidad CEU Cardenal Herrera, Av. Seminario s/n, 46113 Moncada, Valencia Spain

**Keywords:** Herpesvirus, Poxvirus, Walrus, Pinniped

## Abstract

**Background:**

Herpesvirus and poxvirus can infect a wide range of species: herpesvirus genetic material has been detected and amplified in five species of the superfamily *Pinnipedia*; poxvirus genetic material, in eight species of *Pinnipedia*. To date, however, genetic material of these viruses has not been detected in walrus (*Odobenus rosmarus*), another marine mammal of the *Pinnipedia* clade, even though anti-herpesvirus antibodies have been detected in these animals.

**Case presentation:**

In February 2013, a 9-year-old healthy captive female Pacific walrus died unexpectedly at L’Oceanografic (Valencia, Spain). Herpesvirus was detected in pharyngeal tonsil tissue by PCR. Phylogenetic analysis revealed that the virus belongs to the subfamily *Gammaherpesvirinae*. Poxvirus was also detected by PCR in skin, pre-scapular and tracheobronchial lymph nodes and tonsils. Gross lesions were not detected in any tissue, but histopathological analyses of pharyngeal tonsils and lymph nodes revealed remarkable lymphoid depletion and lymphocytolysis. Similar histopathological lesions have been previously described in bovine calves infected with an alphaherpesvirus, and in northern elephant seals infected with a gammaherpesvirus that is closely related to the herpesvirus found in this case. Intracytoplasmic eosinophilic inclusion bodies, consistent with poxviral infection, were also observed in the epithelium of the tonsilar mucosa.

**Conclusion:**

To our knowledge, this is the first molecular identification of herpesvirus and poxvirus in a walrus. Neither virus was likely to have contributed directly to the death of our animal.

## Background

Herpesvirus can infect multiple animal species: mammals, birds, reptiles, fish, frogs and bivalves [1]. The order *Herpesvirales* consists of three families, *Herpesviridae*, *Alloherpesviridae* and *Malacoherpesviridae*, and the *Herpesviridae* family comprises three subfamilies: *Alphaherpesvirinae*, *Betaherpesvirinae* and *Gammaherpesvirinae* [2]. To date, all herpesviruses detected in marine mammals belong to either the *Alpha*- or *Gammaherpesvirinae* subfamilies [3].

Within the order *Carnivora*, in the suborder *Caniformia*, herpesvirus has been described in 11 species [4-10], five of which belong to the superfamily *Pinnipedia*: three form part of the family *Phocidae* [harbor seal (*Phoca vitulina*) [11-14], Hawaiian monk seal (*Monachus schauinslandi*) [15], northern elephant seal (*Mirounga angustirostris*) [16]], and two belong to the family *Otariidae* [California sea lion (*Zalophus californianus*) [17,13,3] and South American fur seal (*Arctocephalus australis*) [18]]. Antibodies against phocine herpesvirus (PhHV) 1 and 2 have been detected in walruses from Alaska and Russia with high seroprevalence [189/341 (55.42%) for PhHV-1, 98/341 (28.74%) for PhHV-2, 61/341 (17.89%) for both viruses in the same sample and 115/341 (33.72%) were negative] [19]. However, we are unaware of studies examining the molecular detection and sequencing of herpesvirus genetic material from walrus.

Poxviruses are pathogens that affect humans and numerous species of wild and domestic animals [20]. While some are species-specific, others can infect a broad species range [21]. Poxvirus has been identified in eight species of the superfamily *Pinnipedia* [22]: five belong to the family *Phocidae* [grey seal (*Halichoerus grypus*) [23-28], harbor seal (*Phoca vitulina*) [29,30,28], Mediterranean monk seal (*Monachus monachus*) [31], spotted seal (*Phoca largha*) [32,33] and Weddell seal (*Leptonychotes weddellii*) [34]], and three belong to the family *Otariidae* [California sea lion (*Zalophus californianus*) [28], Steller sea lion (*Eumetopias jubatus*) [35,32] and South American sea lion (*Otaria flavescens*) [36]]. Some of these pinniped poxviruses have zoonotic potential and can cause lesions in humans, mostly on the skin [23,37].

This article reports the first detection of herpesvirus and poxvirus in a walrus, as well as the first herpesvirus sequences from the family *Odobenidae*.

## Case presentation

An adult female Pacific walrus (*Odobenus rosmarus divergens*, Illiger, 1815), born in June 2003 and housed at L’Oceanografic (Valencia, Spain), died unexpectedly on February 18, 2013. A full necropsy was performed early the next day according to the protocol of the Woods Hole Oceanographic Institution [38]. The animal presented good nutritional condition and moderate postmortem autolysis. Lungs were diffusely dark red and heavy and a clear fluid and foam oozed from a surface cut. No other significant gross lesions were observed.

Representative samples from several organs were collected during necropsy: skin; blubber; muscle; pharyngeal tonsils; thyroid; thymus; tracheobronchial, pre-scapular and mesenteric lymph nodes; lung; heart; stomach; gut; liver; pancreas; spleen; adrenal glands; kidneys; urinary bladder; ovary; genital mucosa; mammary gland; brain; and spinal cord. Three sets of tissue samples and swabs were collected and processed in different ways: one set was used for general bacteriology and stored in transport medium, one was used for virology analysis and stored at −80°C and one was used for conventional histopathology analysis and preserved in 10% neutral buffered formalin.

Formalin-fixed samples were subsequently dehydrated through graded alcohols before being embedded in paraffin wax. Several 4 μm-thick sections were cut from each sample and stained using hematoxylin and eosin.

**Figure 1:**
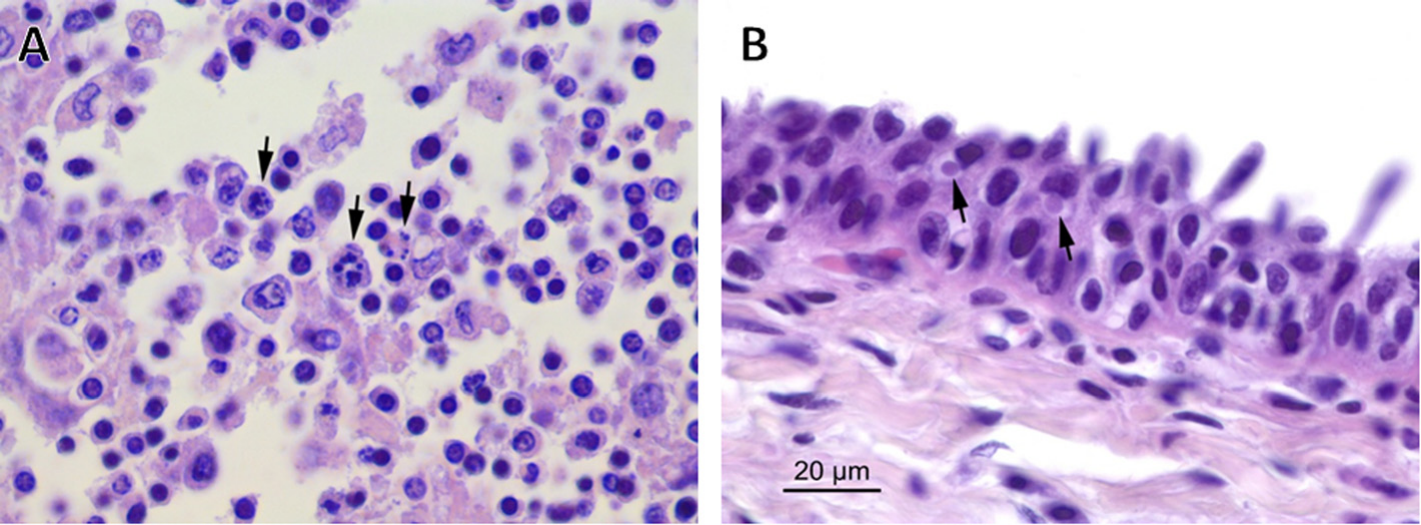
***Odobenus rosmarus divergens*** **.** Pharyngeal tonsil. **(A)** Numerous swollen cells showing condensed chromatin at the periphery of the cell (karyorrhexis) in the germinal center of a lymphoid follicle. **(B)** Intracytoplasmic inclusion bodies (arrow) are observed within epithelial cells. Hematoxylin and eosin.

Lung histology showed diffuse congestion with multifocal hemorrhages. In the alveolar spaces, large amounts of homogeneous eosinophilic material and numerous bacteria were observed in the absence of an associated inflammatory reaction. Pharyngeal tonsils and lymph nodes showed marked lymphoid depletion with lymphocytolysis, which was more evident at the germinal core and was characterized by pyknosis, karyorrhexis and macrophage phagocytosis of cellular debris (Figure 1A). Small round eosinophilic structures were also observed within the cytoplasm of the epithelial cells in the tonsilar mucosa (Figure 1B). These findings were consistent with poxviral intracytoplasmic inclusion bodies. No other findings were observed in any tissue.

For virology analysis, samples of all necropsied tissues were homogenized using a Bullet Blender™ (Next Advance, Averill Park, NY, USA), diluting samples 1:10. DNA of homogenates was extracted using the High Pure Template Preparation Mix (Roche Diagnostics GmbH, Mannheim, Germany), and total RNA was extracted using the NucleoSpin RNA II Kit (Macherey-Nagel, Düren, Germany) following the manufacturers’ instructions.

Samples were analyzed for the presence of herpesvirus, poxvirus and calicivirus.

For herpesvirus detection, a previously described pan-herpesvirus nested polymerase chain reaction (PCR) targeting the DNA polymerase (DNApol) gene was performed for DNA extracts of all sampled tissues [39], and the reactions were positive only for tonsilar tissue. The PCR product was purified by a PCR Purification kit (Qiagen, Germantown, USA) and sequenced to yield a 212-bp sequence, excluding primers (GenBank acc. no. KF972426). Sequence analysis confirmed the herpesvirus diagnosis and revealed the virus to be closely related to gammaherpesvirus. For more detailed virus characterization, a genus-specific PCR for gammaherpesvirus was performed to amplify a region of the glycoprotein B (gB) gene using the set of primers GH1, as previously described [6]. The PCR product was again purified and sequenced, yielding a 453-bp fragment, excluding primers (GenBank acc. no. KF972425).

Phylogenetic analysis of both herpesvirus sequences was carried out using MEGA 5.2 software [40]. In order to evaluate the accuracy of alignments and therefore their capability to produce reliable phylogenetic trees, average amino acid identity was evaluated [41,42]. Average amino acid p-distances (1-amino acid identity) were 0.5068 for the DNApol alignment and 0.4882 for the gB alignment. Since the acceptance threshold was <0.8 for the average p-distance, both sequence alignments were considered adequate. Moreover, in both alignments, each sequence from the databases was compared with the novel sequence from walrus tonsilar tissue in order to calculate the amino acid p-distance between them.

Phylogenetic trees based on amino acid sequences were constructed using MEGA 5.2 with the maximum parsimony (MP) method and the subtree-pruning-and-regrafting (SPR) algorithm [40]. A bootstrap consensus tree from 500 replications was inferred.

**Figure 2:**
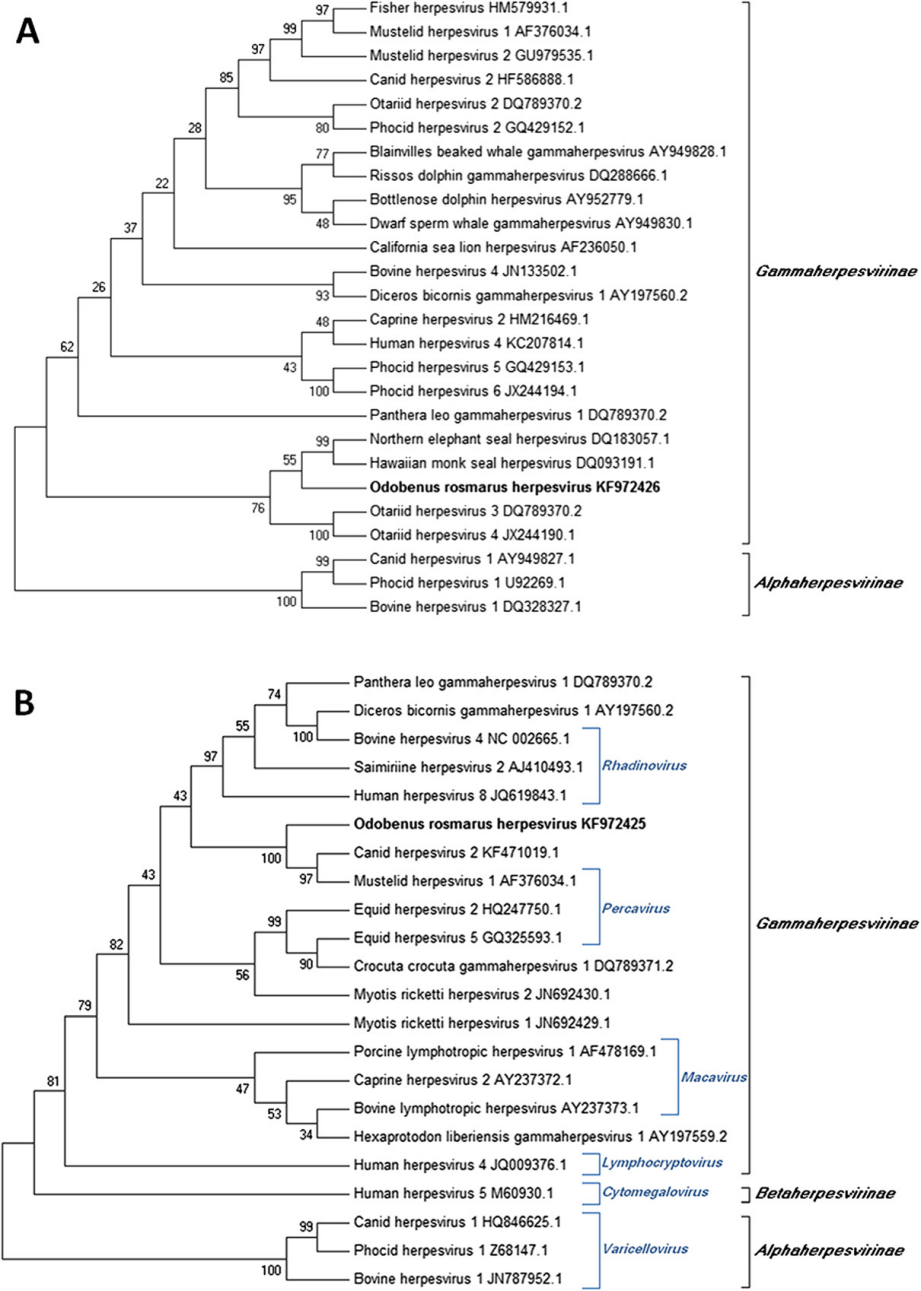
**Phylograms representing the relationships between herpesvirus from** ***Odobenus rosmarus*** **and from other animal species.** Phylogenetic trees were inferred by the maximum parsimony method using the amino acid sequences encoded by the DNA polymerase gene **(A)** and the glycoprotein B gene **(B)**. The results of the bootstrap analysis (500 replications) are indicated at the tree nodes. Each sequence is named according to the virus name and GenBank accession number. *Odobenus rosmarus* herpesvirus is highlighted in bold. The Alpha- **(A, B)** and Betaherpesvirus **(B)** sequences are used as an outgroup in order to root the phylograms. Herpesvirus genera **(B)** are indicated for sequences, as previously assigned by Davison [2].

The phylogenetic analysis revealed that the virus belongs to the subfamily *Gammaherpesvirinae* and suggested that the virus is a member of the genus *Percavirus* (Figure 2). The following sequences in GenBank are most closely related to the herpesvirus DNApol gene sequence from our walrus, based on amino acid p-distances: Hawaiian monk seal herpesvirus [GenBank acc. no. DQ093191.1; amino acid (aa) p-distance 0.2545], northern elephant seal herpesvirus (DQ183057.1; aa p-distance 0.2778) and otariid herpesviruses 3 (DQ789370.2) and 4 (JX244190.1) (nearly identical for this gene region; aa p-distance 0.3818) (Figure 2A). Sequences from the gB gene were unavailable for the four viruses with the most similar DNApol sequences to that of our isolate from walrus, preventing us from analyzing whether the four viruses also encoded similar gB. Among deposited gB gene sequences, the ones closest to our isolate are from mustelid herpesvirus 1 (AF376034.1; aa p-distance 0.1400) and canid herpesvirus 2 (KF471019.1; aa p-distance 0.1467) (Figure 2B). The DNApol gene sequence of *Odobenus rosmarus* herpesvirus showed an aa p-distance of 0.4909 from mustelid herpesvirus 1 and 0.5091 from canid herpesvirus 2.

Phylogenetic branching of herpesvirus resembles that of its hosts [43,13,10]. Consistent with this idea, the DNApol sequences from Genbank most closely related to our isolate are from pinnipeds. Moreover, among the few available gB sequences, the ones most similar to our isolate belong to the same Suborder (*Caniformia*).

In order to determine whether the herpesvirus in our walrus was actively replicating or was in a period of latency at the time of death, the RNA extract from the tonsil sample was used as template in a retrotranscription step with the enzyme Affinity Script QPCR cDNA Synthesis kit (Agilent Technologies, Santa Clara, USA) following the manufacturer’s instructions. The cDNA was used in pan-herpesvirus nested PCR targeting the DNApol gene [39]. Although the PCR clearly gave the expected product, the band in the agarose gel was considerably less intense than that obtained with the DNA sample (data not shown). This result suggests that the herpesvirus was replicating at the time of death in the sampled tonsil tissue as messenger RNA (mRNA) with the herpesvirus sequence was detected.

To evaluate the immune response that our *Odobenus rosmarus* herpesvirus can cause, two direct ELISAs were performed, one for detecting anti-canine herpesvirus immunoglobulin G (IgG) and another for detecting anti-canine herpesvirus immunoglobulin M (IgM). Serum samples were collected from the studied walrus and three other walruses in the captive population of five animals at appoximately 2, 6 and 15 months before the studied animal’s death. The fifth walrus in the population could not be sampled at the same points because it died before the animal under study; instead, we analyzed a serum sample from the fifth animal that had been collected 20 months before the studied animal died. Based on the criterion that positive samples have an optical density of at least 1.00, all samples that we tested were negative for anti-canine herpesvirus IgG.

When the ELISA to detect anti-canine herpesvirus IgM was performed, serum samples from the studied walrus were positive while all remaining samples from other individuals were negative. Given that IgM is the earliest immunoglobulin to be up-regulated after an infection, and no IgG increase was detected in our walrus, it appears that our case was in an early stage of infection involving only a primary immune response at the time of its death.

All tissue samples were tested for the presence of poxvirus using two conventional PCRs targeting the poxvirus DNApol gene or the parapoxvirus DNApol gene, as previously described [32]. Skin and pre-scapular lymph nodes were strongly positive for the desired poxvirus DNApol gene amplicon, while tracheobronchial lymph nodes and tonsil were weakly positive. PCR products were purified and sequenced, yielding a 497-bp sequence from the poxvirus DNApol gene (excluding primers). The viral sequences from the various positive tissues were identical, confirming a diagnosis of poxviral infection.

**Figure 3:**
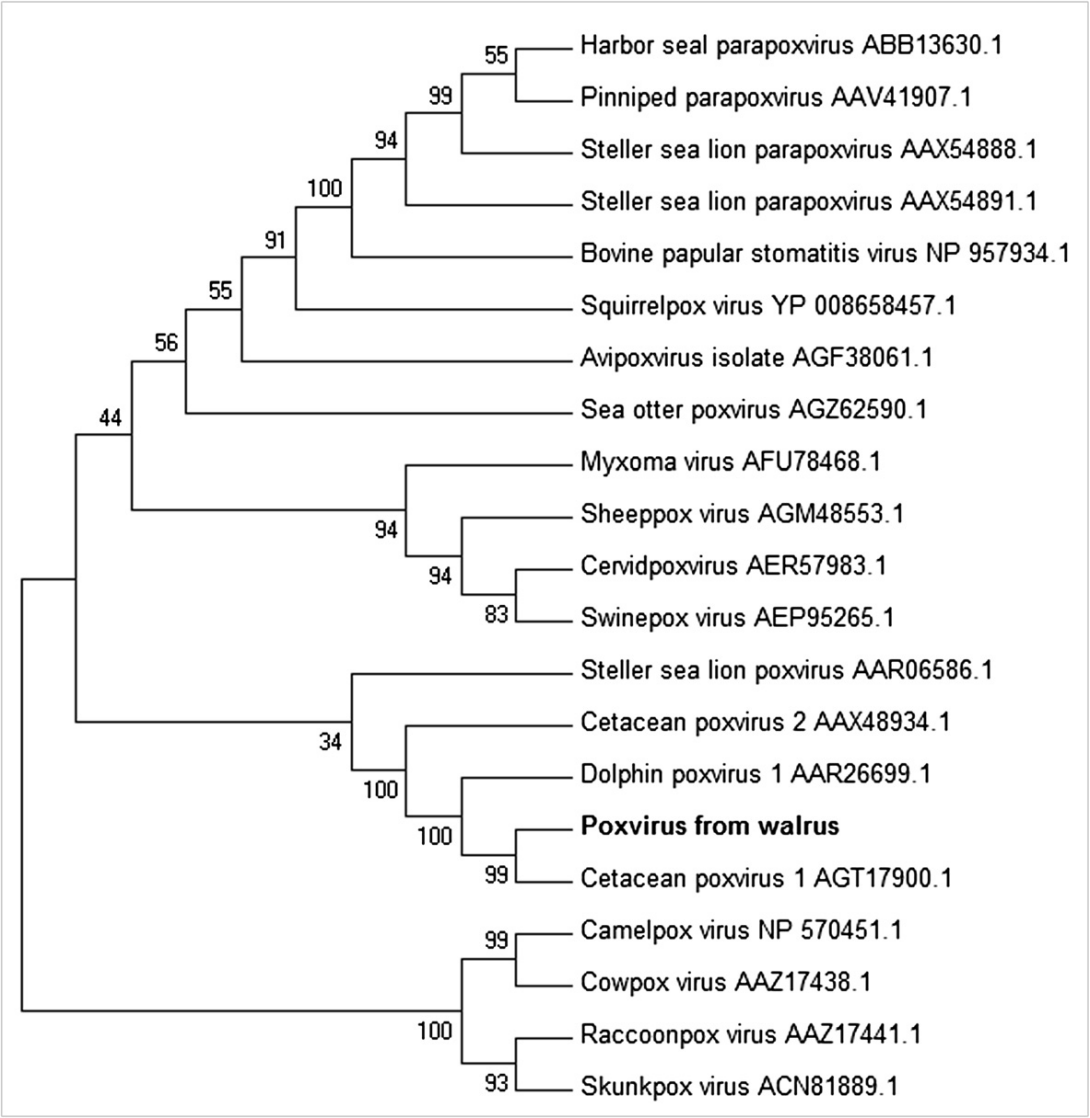
**Phylogram representing relationships between the sequences encoded by the poxvirus DNA polymerase gene from different host species.** This unrooted phylogenetic tree was constructed using the maximum parsimony method based on amino acid sequences. The results of the bootstrap analysis (500 replications) are indicated at the tree nodes. Each sequence name is composed of the virus name and the GenBank accession number. Poxvirus from walrus is highlighted in bold.

Phylogenetic analysis was performed using MEGA 5.2 software, which evaluated average amino acid identity and produced a p-distance of 0.3159. Since this p-distance is still less than 0.8, the approach offers acceptable alignment to produce reliable phylogenetic trees. The phylogenetic tree (Figure 3) was constructed using the MP method equipped with the SPR algorithm and a bootstrap test of 500 replications.

Phylogenetic analysis showed the poxvirus sequence from our walrus to differ by only three nucleotides from the otherwise identical amino acid sequence of a *Cetacean poxvirus 1* from a harbor porpoise (*Phocoena phocoena*) (GenBank KC409049.1) (Figure 3). In order to verify whether this result was due to sample contamination, poxvirus analysis in the walrus samples was repeated twice independently; DNA was homogenized and extracted with no positive controls, and the same result was obtained as described above. This striking sequence similarity between cetacean poxvirus 1 and our walrus isolate suggests that this poxvirus may have a broad host range. The walrus in our study lived in the same building with two beluga whales (*Delphinapterus leucas*). Although the water and enclosures were different for both species and direct contact was impossible, the trainers were the same, suggesting that they may be the source of poxvirus cross-contamination among species. Poxviruses are enveloped viruses and most can persist in the environment for long periods of time [44-46], which increases the risk of viral spread via fomites. The mechanical transport of viruses by trainers is possible. In addition, some marine mammal poxviruses have been described as zoonotic [23,37], and in this case, the virus may have also infected the trainers and remained latent or replicated slowly, without causing injury.

To test this hypothesis, poxvirus was determined in samples of healthy skin, skin lesions and blood from the two beluga whales. These animals shared the same facility with the walrus during almost 8 years. Oral swabs from the three trainers were collected using the same protocol as for the walrus samples. All samples were negative for cetacean poxvirus. From this analysis of only two tissues at a single point in time, we cannot rule out beluga whales as the source of infection, especially since the animals shared the same building for 8 years, so transmission could have occurred at any point. All the animals were housed at the Oceanografic of Valencia, and all the national and international permits were in order. No special permits were required for this research because serum samples from the walruses (antibodies against herpesvirus determination) and blood and skin samples from the beluga whales (poxvirus determination) were performed within the Oceanografic’s routine procedures.

In order to complete the viral diagnosis, RNA of all the samples was tested for calicivirus by real-time PCR as described [47], and all samples were negative for calicivirus determination.

General bacteriology analysis of different tissue samples did not identify any potential pathogens; some bacteria were found but these were thought to be either components of the normal bacterial flora or post mortem contaminants. These results are consistent with histopathological findings, which detected no tissue damage or inflammatory reaction associated with *in vivo* bacterial infection.

Although the histological findings observed in pharyngeal tonsil and lymph nodes were nonspecific, lymphoid depletion and lymphocytolysis have previously been associated with viral infections in other mammals, such as in calves experimentally infected with alphaherpesvirus [48,49], or northern elephant seals infected with gammaherpesvirus [16], which are closely related phylogenetically to the *Odobenus rosmarus* herpesvirus reported here (Figure 2A). Although no typical intranuclear inclusion bodies were observed in the examined organs, herpesviral inclusion bodies are known to be frequent in the very early stages of infection, but they are rarely observed beyond day 7 post-infection [50].

Based on clinical history and necropsy findings, the sudden death of this walrus was likely due to drowning and not to the herpesvirus or poxvirus infections described in this paper.

## Conclusions

Histopathology and identification and phylogenetic characterization of herpesvirus and poxvirus in our case, together with the presence of RNA with the herpesvirus sequence, indicate that gammaherpesvirus is the most probable etiologic agent responsible for the microscopic lesions of the tonsil and lymph nodes, and that poxvirus is the most likely cause of the intracytoplasmic inclusion bodies found in the tonsil mucosa. To the best of our knowledge, this report describes the first detection and characterization of herpesvirus and poxvirus in walrus.

## Abbreviations

PCR: Polymerase chain reaction
PhV: Phocine herpesvirus
DNApol: DNA polymerase
gB: Glycoprotein B
MP: Maximum parsimony
SPR: Subtree-pruning-and-regrafting
aa: Amino acid
IgG: Immunoglobulin G
IgM: Immunoglobulin M
